# Sphingosine-1-Phosphate Induces Dose-Dependent Chemotaxis or Fugetaxis of T-ALL Blasts through S1P1 Activation

**DOI:** 10.1371/journal.pone.0148137

**Published:** 2016-01-29

**Authors:** Carolina V. Messias, Eliane Santana-Van-Vliet, Julia P. Lemos, Otacilio C. Moreira, Vinicius Cotta-de-Almeida, Wilson Savino, Daniella Arêas Mendes-da-Cruz

**Affiliations:** 1 Laboratory on Thymus Research, Oswaldo Cruz Institute, Oswaldo Cruz Foundation, Rio de Janeiro, Rio de Janeiro, Brazil; 2 Laboratory of Molecular Biology and Endemic Diseases, Oswaldo Cruz Institute, Oswaldo Cruz Foundation, Rio de Janeiro, Rio de Janeiro, Brazil; University of Birmingham, UNITED KINGDOM

## Abstract

Sphingosine-1-phosphate (S1P) is a bioactive sphingolipid involved in several physiological processes including cell migration and differentiation. S1P signaling is mediated through five G protein-coupled receptors (S1P1-S1P5). S1P1 is crucial to the exit of T-lymphocytes from the thymus and peripheral lymphoid organs through a gradient of S1P. We have previously observed that T-ALL and T-LBL blasts express S1P1. Herein we analyzed the role of S1P receptors in the migratory pattern of human T-cell neoplastic blasts. S1P-triggered cell migration was directly related to S1P1 expression. T-ALL blasts expressing low levels of S1P1 mRNA (HPB-ALL) did not migrate toward S1P, whereas those expressing higher levels of S1P1 (MOLT-4, JURKAT and CEM) did migrate. The S1P ligand induced T-ALL cells chemotaxis in concentrations up to 500 nM and induced fugetaxis in higher concentrations (1000–10000 nM) through interactions with S1P1. When S1P1 was specifically blocked by the W146 compound, S1P-induced migration at lower concentrations was reduced, whereas higher concentrations induced cell migration. Furthermore, we observed that S1P/S1P1 interactions induced ERK and AKT phosphorylation, and modulation of Rac1 activity. Responding T-ALL blasts also expressed S1P3 mRNA but blockage of this receptor did not modify migratory responses. Our results indicate that S1P is involved in the migration of T-ALL/LBL blasts, which is dependent on S1P1 expression. Moreover, S1P concentrations in the given microenvironment might induce dose-dependent chemotaxis or fugetaxis of T-ALL blasts.

## Introduction

Sphingosine-1-phosphate (S1P) is a membrane-derived lipid produced by mast cells, endothelial cells [1], pericytes [2] and especially by activated platelets and erythrocytes [3]. This lipid is produced by an enzymatic cascade of sphingolipids through phosphorylation of sphingosine by sphingosine kinase 1 or 2 (SphK1 and SphK2) [4, 5]. S1P is involved in several physiological processes in the immune, cardiovascular and nervous systems, including cell proliferation, survival, migration and differentiation, angiogenesis, inflammation and calcium homeostasis [6, 7]. Furthermore, S1P is involved in tumor progression [8], neoplastic cell proliferation [9–11], migration [12, 13] as well as resistance to chemotherapeutic drugs [14, 15].

S1P signaling is primarily mediated through five G protein-coupled receptors (S1P1-S1P5). S1P1, originally named EDG-1, was the first S1P receptor described and is the only S1P receptor exclusively coupled to G_i_, being ubiquitously expressed. Its major functions are related to vascular development and integrity, and to the mobility of different hematopoietic cells types (hematopoietic progenitors, T and B lymphocytes, natural killer T cells, dendritic cells, macrophages, neutrophils, mast cells and osteoclasts) [7, 16]. This mobility is associated with a gradient of S1P since this lipid is found in higher concentrations in the blood and in lower amounts within lymphoid organs [3, 17].

S1P1 is crucial to the exit of T lymphocytes from the thymus and peripheral lymphoid organs [18, 19]. Mouse double-positive immature thymocytes (CD4^+^CD8^+^) express relatively low levels of S1P1, as compared with single-positive mature thymocytes (CD4^+^CD8^-^ or CD4^-^CD8^+^) [18]. Single-positive thymocytes migrate toward S1P, a response that is no longer seen in thymocytes deficient in S1P1. Actually, S1P1-deficient thymocytes are able to differentiate in the thymus but are not able to leave the thymus, thus accumulating within the organ [18, 19]. Moreover, mature T lymphocytes deficient in S1P1 are not seen in the blood or peripheral organs, and transplantation of these cells to normal mice leads to their accumulation in peripheral organs [19]. These data support the hypothesis that S1P1 is one of the major players in the exit of T lymphocytes from lymphoid organs.

Similar to what has been seen in mice [18], we have recently reported that normal human double-positive thymocytes express less S1P1 than CD4 and CD8 single-positive thymocytes. Moreover, human thymocytes migrate toward S1P according to the expression levels of S1P1. This response is associated with early step events of cell migration such as actin polymerization. Interestingly, S1P1 is also expressed by human malignant T-cell precursors. Indeed, T-cell acute lymphoblastic leukemia (T-ALL) and T-cell lymphoblastic lymphoma (T-LBL) blasts express similar levels of S1P1 and migrate toward S1P [20]. As T-ALL/LBL present similar characteristics of normal T-lymphocyte precursors [21], they might also share mechanisms of migration and homing through S1P receptors. Considering the importance of T-ALL/LBL migration and spread during disease pathogenesis, we analyzed herein the role of different S1P concentrations in the migratory response of human T-cell lymphoblasts and the signaling pathways involved in S1P1 activation.

## Materials and Methods

### Antibodies and chemicals

Sphingosine-1-phosphate, W146, (4R)-2-Undecyl-4-thiazolidinecarboxylic acid (BML-241), FITC-phalloidin, L-alpha-phosphatidylcholine palmitoyl and fatty acid free bovine serum albumin (BSA) were obtained from Sigma-Aldrich (St Louis, USA). Rabbit anti-phospho-Akt (catalog number: 9271), rabbit anti-Akt (catalog number: 4685), rabbit anti-p44/42 MAPK (catalog number: 4695), rabbit anti-phospho-p44/42 MAPK (catalog number: 9101) and goat anti-rabbit HRP-linked (catalog number: 7071) polyclonal or monoclonal antibodies were purchased from Cell Signaling (Danvers, USA), whereas the mouse anti-actin monoclonal antibody (clone C4—catalog number: MAB1501) was from Merck Milipore (Darmstadt, Germany) and the goat anti-mouse HRP-linked antibody (catalog number: 1031–05) was from Southern Biotech (Birmingham, USA).

### Cell lines

T-cell acute lymphoblastic leukemia (HPB-ALL, MOLT-4, JURKAT, CEM) [22–25] and anaplasic large lymphoma (SU-DHL-1) [26] were kindly provided by Dr. Vahid Asnafi (Assistance Publique-Hopitaux de Paris, Hopital Necker-Enfants Malades, Paris, France). Cell lines were cultured in RPMI-1640 (Sigma-Aldrich), pH 7–7.5, supplemented with 10% fetal bovine serum (Cultilab, Campinas, Brazil), 2 g/L sodium bicarbonate (Isofar, Rio de Janeiro, Brazil), 2 g/L HEPES (Sigma-Aldrich), 2 mg/ml ciprofloxacin at 37°C in an atmosphere containing 5% of CO_2_. MOLT-4 was the only cell line supplemented with 20% fetal bovine serum.

### Real-time quantitative polymerase chain reaction (RQ-PCR)

mRNA was isolated from cells using RNase Mini Kit following the manufacturer’s instructions (Quiagen, Hilden, Germany). cDNA synthesis was done with 2 μg of mRNA with Super Script II RT (Invitrogen, Carlsbad, USA). S1P1 and ABL primers were designed using the Primer Express^®^ software (Applied Biosystems, Foster City, USA). S1P2, S1P3, S1P4 and S1P5 primers were obtained from the literature [27]. S1P1 forward: GGCTCTCCGAACGCAACTT; S1P1 reverse: CAGGCTTTTTGTGTAGCTTTTCC; S1P2 forward: GCCATTGTGGTGGAAAACCTT; S1P2 reverse: CAGGTTGCCCAGAAACAGGTA; S1P3 forward: AGCGGCACTTGACAATGATCA; S1P3 reverse: ACATCCCGATCAGGAGGAAGA; S1P4 forward: CCCTCTACTCCAAGCGCTACA; S1P4 reverse: CCATAGAGGCCCATGATGGT; S1P5 forward: TGAAGGAGTAGTTCCCGAAGG; S1P5 reverse: AAGCTTCTATGGCTCCCACCTC; ABL forward: TGGAGATAACACTCTAAGCATAACTAAAGGT; ABL reverse: GATGTAGTTGCTTGGGACCCA. RQ-PCR was performed with Syber^®^ Green PCR Master Mix (Applied Biosystems) on Step One Plus System (Applied Biosystems). Results were normalized by the gene control ABL, which is recommended by Europe Against Cancer Program for leukemia’s diagnosis [28]. Fold change analysis were done using HPB-ALL as calibrator and statistical analysis was made with ΔCt values.

### Cell migration assays

Cells were serum-starved for 2 h and then treated or not with 100 μM of W146 (Sigma-Aldrich) and/or 100 μM BML-241 (Sigma-Aldrich) for 1 h at 37°C and 5% CO_2_ atmosphere. This treatment (using 2 x 10^6^ cells per well) was performed in 100 μl of RPMI- fatty acid free BSA 0.1%. Migration assays were done in transwell chambers. Cell culture inserts of 8μm pore size (Nunc, Roskilde, Denmark or Corning Costar, Cambridge, USA) were treated with PBS- fatty acid free BSA 0.1% for 45 min at 37°C under 5% CO_2_ atmosphere. For chemotaxis assays, 2 x 10^6^ cells in 300 μl (inserts from Nunc) of migration medium (RPMI- fatty acid free BSA 0.1%) were added in the upper chambers and 500 μl of the migration medium alone or containing S1P were added in the lower chamber. For fugetaxis assays, 2 x 10^6^ cells in 100 μl (inserts from Corning Costar) of migration medium alone or containing S1P were added to the upper chamber and 600 μl of migration medium alone were added to the lower chamber. For checkerboard assays, 2 X 10^6^ cells in 100 μl (inserts from Corning Costar) of migration medium alone or containing S1P were added to the upper chamber and 600 μl of migration medium alone or containing S1P were added to the lower chamber. Cells were allowed to migrate for 4 h and the numbers of migrating cells in the lower chamber were counted using a Neubauer chamber. In some experiments, cells in the upper chamber were also collected and stained with Anexin-V-FITC and PI and viability was evaluated by flow cytometry in a FACSCanto™ II device (Becton Dickinson, San Jose, USA).

### Actin polymerization assays

Cells were serum-starved for 2 h and then treated or not with 100 μM W146 for 1 h as detailed above. After treatment, cells were incubated in RPMI/20 mM HEPES and S1P was added. At each indicated time point (15 s to 2 min), an aliquot was taken from the cell suspension and mixed with the labeling buffer, consisting in 10^−7^ M FITC-phalloidin, 0.125 mg/ml L-alpha-phosphatidylcholine palmitoyl, and 4.5% paraformaldehyde in PBS. Staining was analyzed by flow cytometry, and the mean fluorescence intensity (MFI) values obtained before addition of the ligand were arbitrarily set at 100%.

### Cell stimulation

Serum-starved cells (2 x 10^6^) were centrifuged and ressuspended in RPMI- fatty acid free BSA 0.1% and S1P was added to the cell suspension and at each time point (1 to 60 min) and then washed with cold PBS.

### Western Blot

Protein extraction was performed in 70 μl of protease and phosphatase cocktail (Sigma-Aldrich) with aid of a 1 ml syringe coupled to a needle immediately after cell stimulation. Total protein content was determined in Qubit fluorometer (Invitrogen) following manufacturer's recommendations. Proteins were resolved by Bolt 4–12% Bis-Tris Plus Gel (Novex, Carlsbad, USA) and transferred by iBlot® Dry Blotting System (Invitrogen) to nitrocellulose membranes. Membranes were blocked with 5% non-fat milk in tris-buffered saline with tween 20 (TBST) for 2 h at room temperature. The membranes were then labeled with anti-AKT, anti-pAKT, anti-p44/42 MAPK or anti-phospho-p44/42 antibodies (1:1000) diluted in 2,5% non-fat milk in TBST for 2 h at room temperature. After incubation, membranes were washed twice for 10 min and twice for 5 min with TBST. Peroxidase-conjugated anti-rabbit or anti-mouse IgG were used as secondary antibodies (1:1000) and samples were incubated for 90 min at room temperature. Subsequently, membranes were washed again, incubated with Enhanced Chemiluminescence (ECL) Western Blotting Detection Reagents (Amersham, Buckinghamshire, UK) for 3 min at room temperature and exposed to Hyperfilm ECL (Amersham). When necessary, brightness and contrast adjustments were performed in the entire images.

### Rac1 Activation assays

Rac-1 activity was assessed with Rac1 G-LISA Activation Assay Kit (Cytoskeleton, Inc., Denver, USA). Protein extraction and quantification were performed according to the manufacturer's instructions immediately after cell stimulation. Rac activation signal was measured as absorbance levels using 490 nm filter. Optical density (OD) values obtained before addition of the ligand were arbitrarily set at 100%.

### Statistical analyses

Results were analyzed by unpaired Student’s *t* test, One-way ANOVA followed by Tukey or Dunnett post-test or Two-way ANOVA followed by Bonferroni post-test in GraphPad Prism 5. Differences were considered to be statistically significant when p<0.05 (* or #), p<0.01 (** or ##) or p<0.001 (*** or ###).

## Results

### S1P receptors are expressed in T-ALL blasts

**Fig 1:**
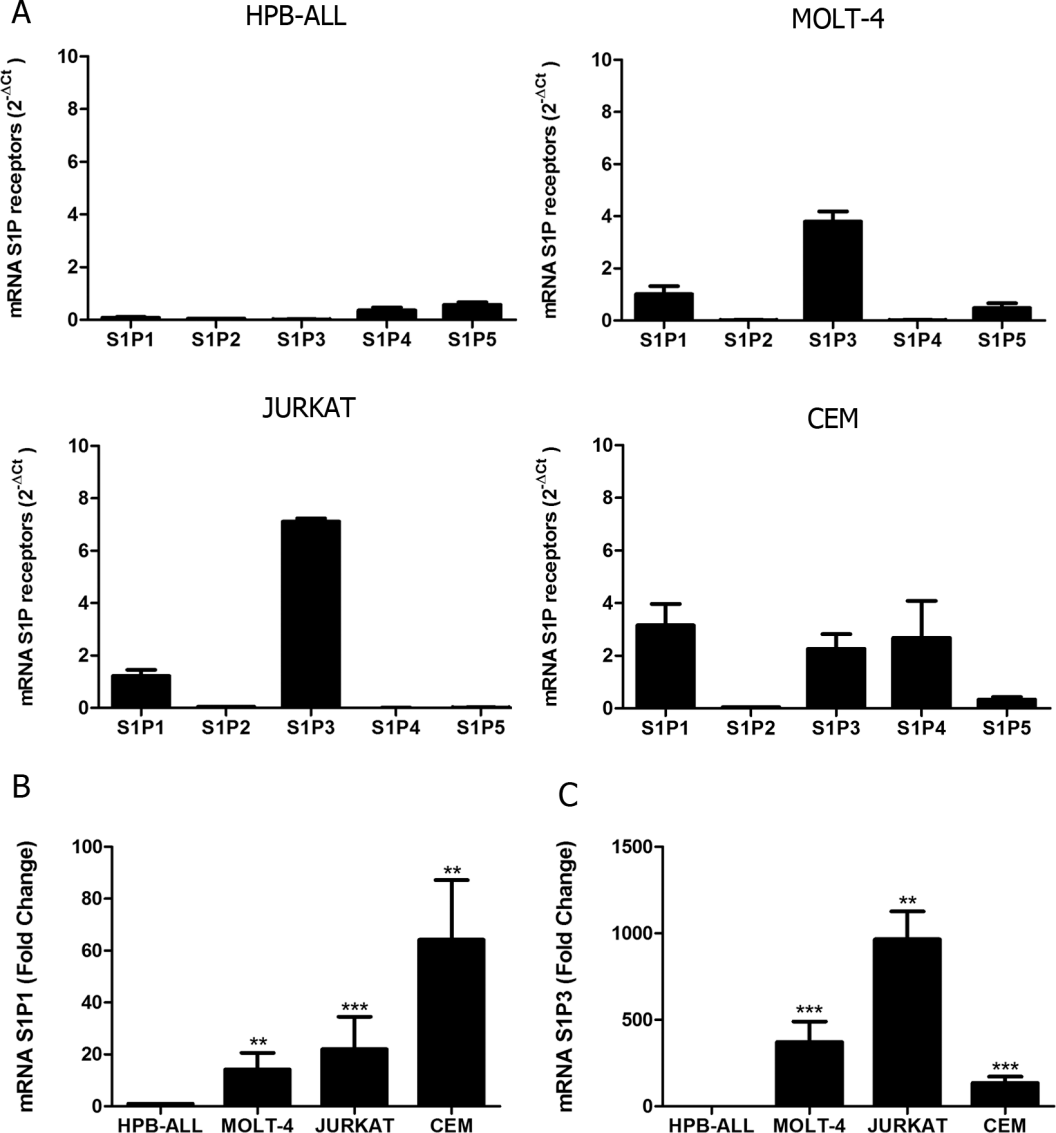
S1P receptors mRNA expression in human T-ALL blasts. **(A)** Bars show S1P receptors mRNA expression analyzed by real time quantitative PCR, compared with the control Abelson (Abl) gene (2^-ΔCt^) in T-ALL (n = 1–2, with 3–6 biological replicates). **(B)** Bars shows S1P1 or (C) S1P3 mRNA expression analyzed by real time quantitative PCR, compared with the control Abelson (Abl). Fold change analysis were done using HPB-ALL as calibrator to normalize the expression of the receptors on the other T-ALL blasts. Statistical analysis was made with ΔCt values and significant differences are related to HPB-ALL cells. Results are expressed as mean ± SEM and were analyzed by Student’s *t* test and differences were considered statistically significant when p<0.05 (*), p< 0.01 (**) or p< 0.001(***) (n = 1–2, with 3–6 biological replicates).

We first analyzed gene expression of all S1P receptors (S1P1-S1P5) in different T-ALL blasts by quantitative RT-PCR. We detected variable levels of the S1P receptors in the four cell lines studied. We observed that HPB-ALL cells mainly expressed S1P4 and S1P5 mRNA, whereas MOLT-4 cells showed a more intense expression of S1P1, S1P3 and S1P5. JURKAT cells preferentially expressed genes coding for S1P1 and S1P3. Except for S1P2, CEM cells expressed all other S1P receptors (Fig 1A).

Among S1P receptors, S1P1, S1P2 and S1P3 are the most studied. S1P1 and S1P3 are involved in inducing cell motility, while S1P2 is involved in the inhibition of this process [29]. As HPB-ALL expressed very low levels of S1P1 and S1P3 we used this cells in order to normalize the expression of the receptors on the other T-ALL cell lines. We observed that S1P1 gene expression in MOLT-4, JURKAT and CEM cells was, respectively, 14, 22 and 64 times higher than in HPB-ALL cells (Fig 1B). Regarding S1P3, its expression was, respectively, 372, 965 and 136 times higher than HPB-ALL cells (Fig 1C).

### Low S1P concentrations induced chemotactic migration of T-ALL blasts

**Fig 2:**
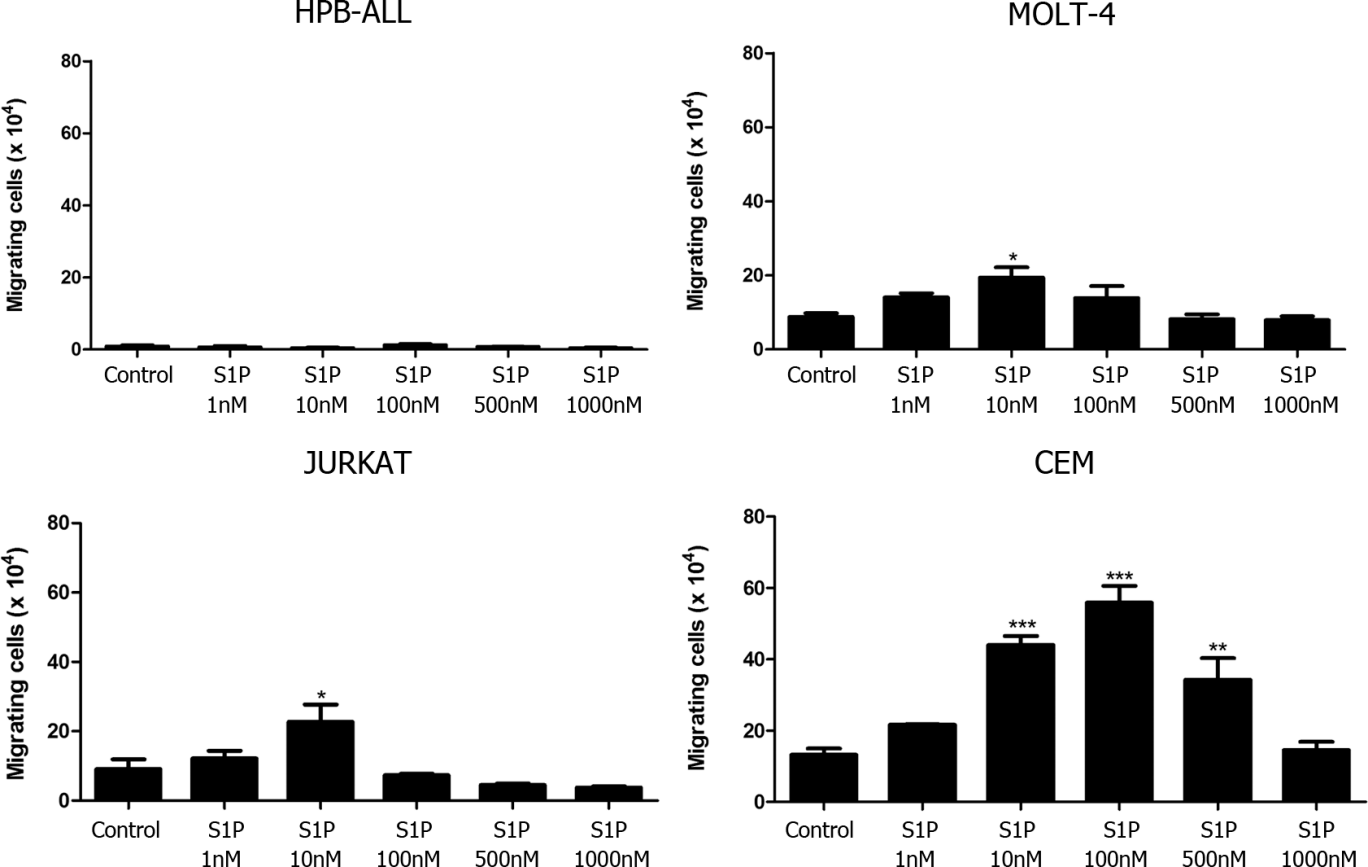
Different S1P concentrations induce chemotactic responses of T-ALL blasts. T-ALL blasts were serum-starved for 2 h and inserted into Transwell™ chambers with different S1P concentrations for 4 hours. Results are expressed as mean ± SEM and were analyzed by One-way ANOVA, followed by Tukey post-test. Differences were considered statistically significant when * p˂0.05, ** p ˂0.01 or *** p ˂0.001 (n = 3).

Functionally, we evaluated whether different concentrations of S1P could act as chemoattractant stimulus upon T-ALL cell lines. We observed that HPB-ALL cells, which express very low S1P1 mRNA levels, did not migrate toward S1P in any concentration used (1–1000 nM–Fig 2). Differently, MOLT-4 and JURKAT cells, which express comparably moderate S1P1 amounts, migrated at low rates toward S1P 10 nM, but failed to migrate toward higher concentrations (500 and 1000 nM). CEM cells, which expressed higher S1P1 levels, migrated at a higher rate and respond to a wider concentration range of S1P (10, 100 and 500 nM) than MOLT-4 and JURKAT cells (Fig 2). When we compared the S1P-induced migration rate (10 nM) of the various T-ALL cells as a function of the corresponding S1P1 gene expression level, we noticed that the numbers of migrating cells were higher for CEM cells and lower for MOLT-4 and JURKAT, suggesting that S1P1 mRNA expression is directly correlated with their migration capacity (S1 Fig).

### S1P1 is involved in S1P-induced migration of T-ALL blasts

**Fig 3:**
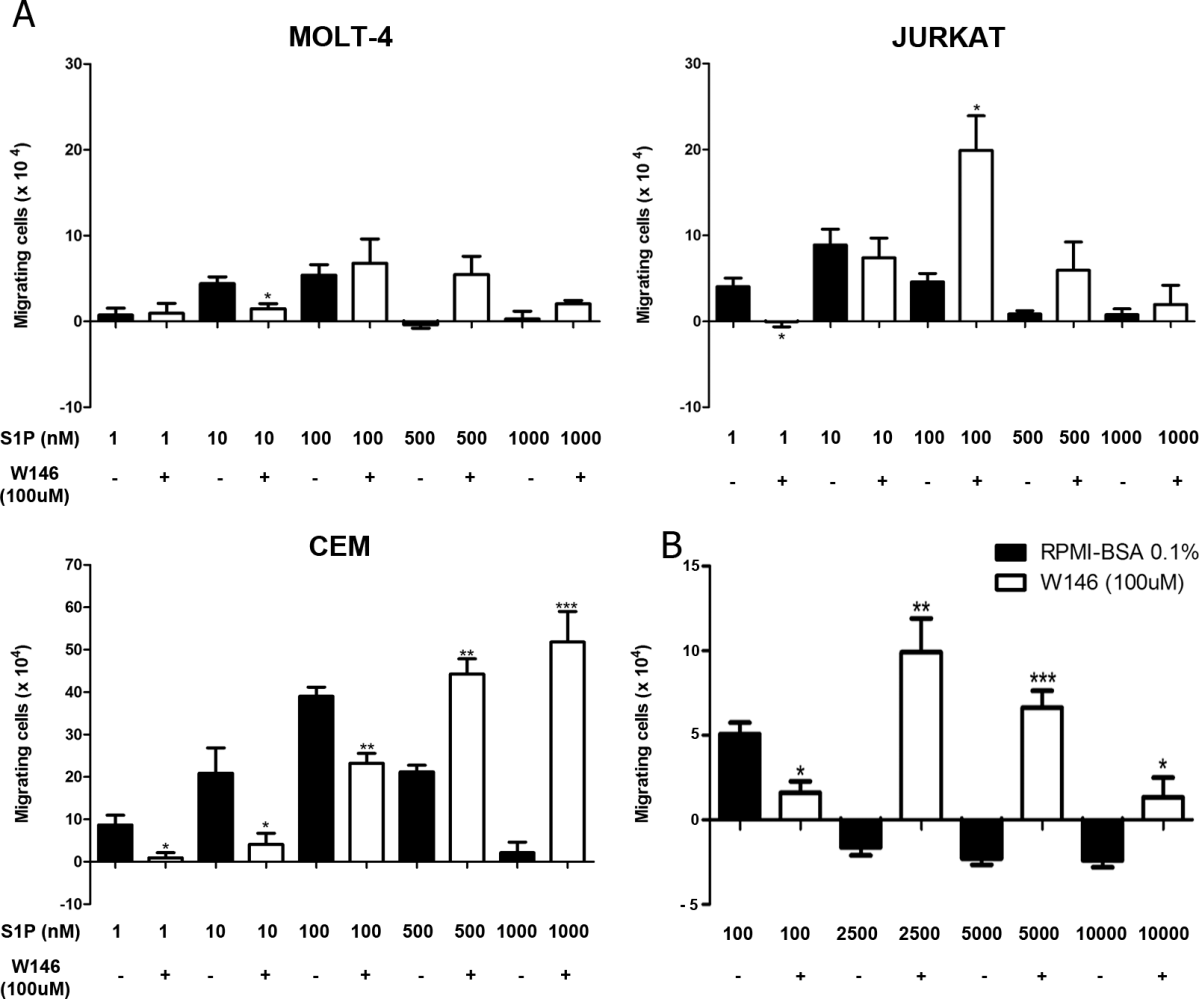
S1P1 is involved in S1P-driven chemotactic responses of T-ALL blasts. T-ALL blasts were serum-starved for 2 h and pre-treated or not with W146 (100 μM). **(A)** Cells were applied to Transwell™ chambers with S1P 1, 10, 100, 500 or 1000 nM and incubated for 4 hours (n = 3). **(B)** Migratory response of CEM cells toward S1P 100, 2500, 5000 and 10000 nM (n = 3). Values correspond to a specific migration after subtracting the numbers of migrating cells obtained in wells with culture medium only. Black bars correspond to T-ALL blasts pre-treated with RPMI-BSA 0.1% and white bars correspond to T-ALL blasts pre-treated with W146. Results are expressed as mean ± SEM and were analyzed by unpaired Student’s *t* test. Differences were considered statistically significant when * p˂0.05, ** p ˂0.01 or *** p ˂0.001.

To investigate if the S1P-evoked migration occurred due to specific interactions between S1P/S1P1, cells were pre-treated with the W146 compound, a S1P1 specific inhibitor [30–32]. Such pre-treatment inhibited the migration of MOLT-4 cells toward S1P 10 nM, whereas pre-treatment of JURKAT cells significantly inhibited migration toward S1P 1 nM, but not 10 nM. In the case of CEM cells, W146 blocked the migration in various S1P concentrations, namely 1, 10 and 100 nM (Fig 3A). These results suggest that migration induced by low concentrations of S1P in T-ALL blasts is specifically mediated by S1P1.

Unexpectedly, JURKAT cells pre-treated with W146 migrated efficiently toward S1P 100 nM, but much less toward higher concentrations, such as 1000 nM. After CEM cell pre-treatment with the S1P1 blocker, we observed that S1P 500 and 1000 nM strongly induced cell migration (Fig 3A). We then enhanced S1P concentrations to investigate if even at higher concentrations, CEM cells pre-treaded with W146 would still be able to migrate. As expected, we observed that S1P 2500, 5000 and 10000 nM did not induce cell migration. However, CEM cells pre-treated with W146 migrated toward S1P applied at 2500 and 5000 nM, but did not migrate when exposed to S1P 10000 nM (Fig 3B), where migration response returned to basal levels. Similar effect was observed with MOLT-4 cells pre-treated with W146, even though differences between pre-treated and non-treated cells were not statistically different for higher S1P concentrations, such as 100–1000 nM (Fig 3A). Together, our data indicate that the pretreatment of the T-ALL blasts with W146 shifted the S1P dose/chemoattraction response curve. Indeed, the maximum chemotaxis was reached at approximately one-log higher S1P dose, following W146 pre-treatment.

### S1P1 mediates fugetaxis of T-ALL at high S1P concentrations

**Fig 4:**
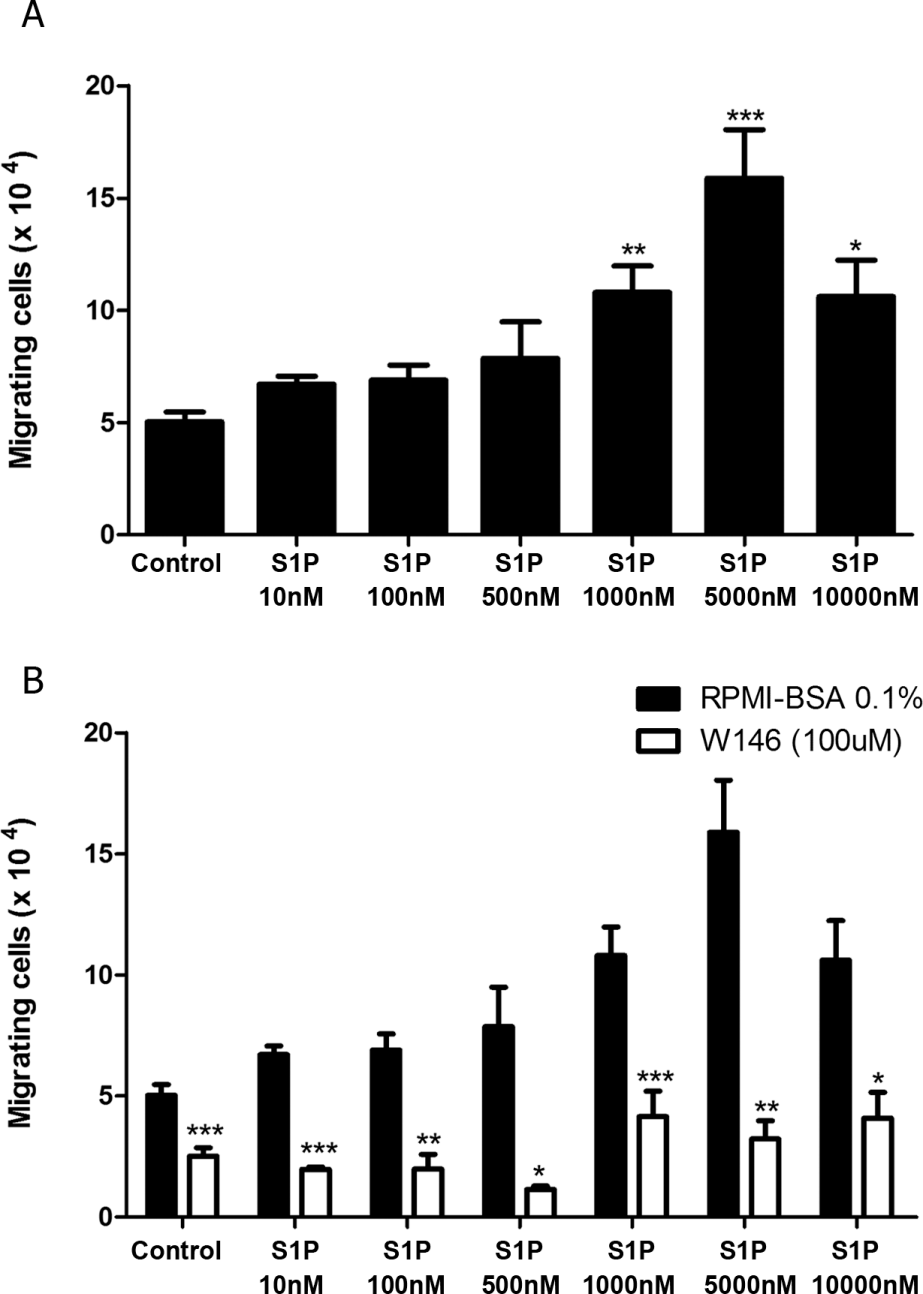
High S1P concentrations induce S1P1-dependent fugetaxis of CEM cells. **(A)** CEM cells were serum-starved for 2 h, applied to Transwell™ chambers containing different S1P concentrations and incubated for 4 hours. S1P was added to the upper chamber to evaluate fugetaxis; only RPMI-BSA 0.1% was added to the bottom chambers. Results were analyzed by One-way ANOVA, followed by Tukey post-test (n = 3). **(B)** CEM cells were serum-starved for 2 h and pre-treated or not with W146 (100 μM). Cells were then applied to Transwell™ chambers containing different S1P concentrations and incubated for 4 hours. S1P was added to the upper chamber to evaluate repulsive responses. Black bars correspond to T-ALL blasts pre-treated with RPMI-BSA 0.1% alone and white bars correspond to T-ALL blasts pre-treated with W146. Results were analyzed by unpaired Student’s *t* test. Results are expressed as mean ± SEM and differences were considered statistically significant when * p˂0.05, ** p ˂0.01 or *** p ˂0.001 (n = 3).

Since we verified that high S1P concentrations failed to induce chemotaxis of T-ALL blasts, we then evaluated whether these concentrations were actually repulsive. We performed migration assays where different S1P concentrations were applied to the transwell upper chambers together with CEM cells, which express higher levels of S1P1. We found that high S1P concentrations (1000, 5000 and 10000 nM) induced fugetaxis or cell chemorepulsion, whereas lower concentrations did not (Fig 4A). The most significant effect was observed at S1P 5000 nM. This chemorepulsive effect was also S1P1 dependent, since pre-treatment of cells with W146 inhibited fugetaxis responses induced by S1P 1000, 5000 and 10000 nM (Fig 4B). Interestingly, W146 also inhibited migratory responses from control and lower S1P concentrations (10, 100 and 500 nM).

**Fig 5:**
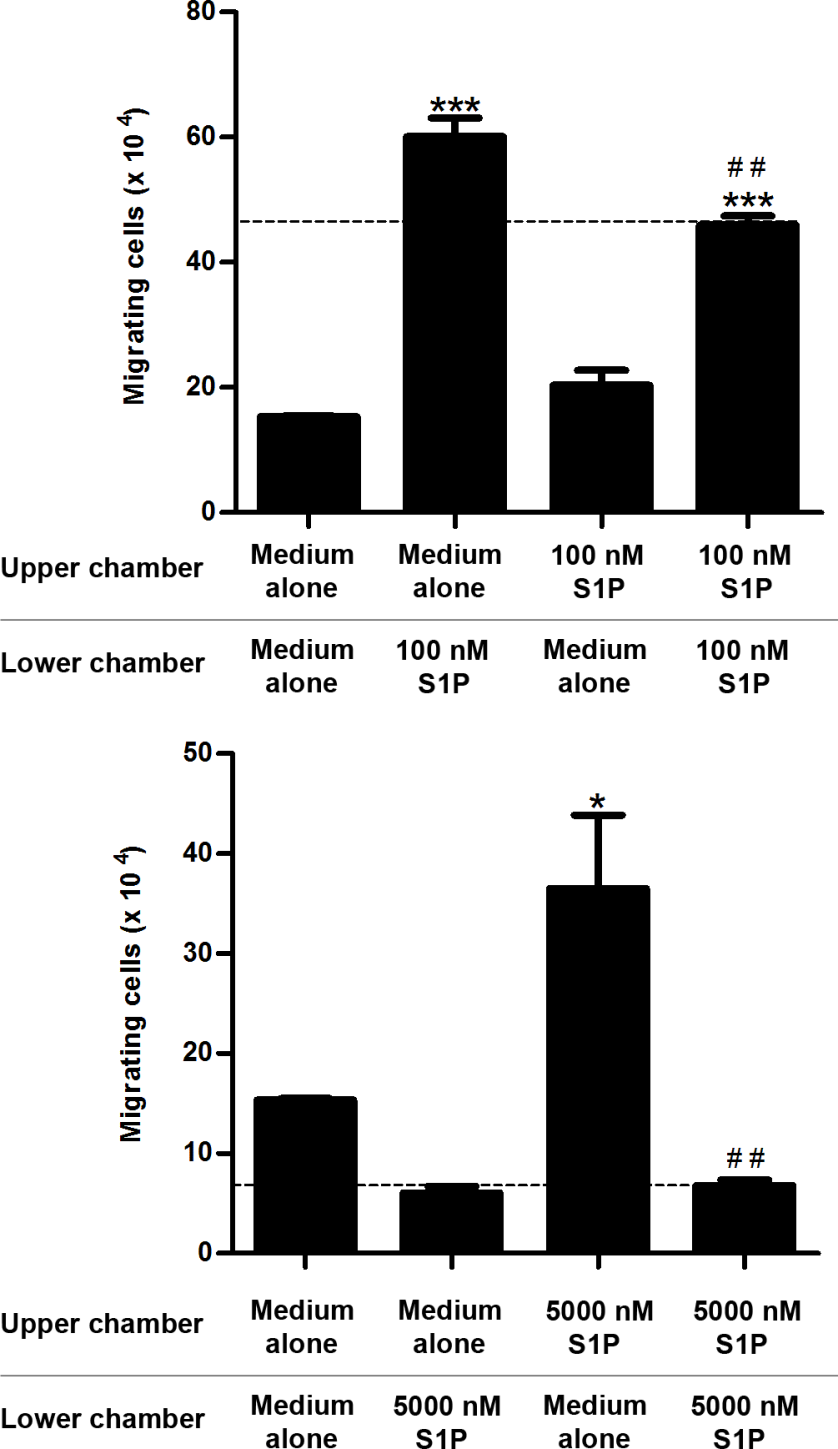
S1P-induced migratory responses in checkerboard assays. CEM cells were serum-starved for 2 h, applied to Transwell™ chambers containing different S1P concentrations in a checkerboard format and incubated for 4 hours. **(A)** S1P 100 nM or **(B)** S1P 5000 nM were added to upper and/or lower chamber as RPMI-BSA 0,1%. Results were analyzed by One-way ANOVA, followed by Tukey post-test and are expressed as mean ± SEM. Differences between wells with medium alone in upper and lower chambers and wells with S1P in upper and/or lower chamber were considered statistically significant when * p˂0.05, ** p ˂0.01 or *** p ˂0.001. Differences between wells with S1P in lower (100 nM) or upper (5000 nM) chambers and wells with S1P in both upper and lower chambers were considered statistically significant when # p˂0.05, ## p ˂0.01 or ### p ˂0.001 (n = 3).

To confirm that different S1P concentrations induce fugetaxis or chemotaxis, we performed checkerboard assays. We observed that low S1P concentrations (100 nM) induced chemokinesis, as cell migration was observed when S1P was added to both upper and lower transwell™ chambers, (Fig 5A). However, chemotaxis was still induced by S1P 100 nM, since migratory response induced by S1P added only to the lower chamber was higher than migratory response observed when S1P was present on both chambers (Fig 5A). In contrast, higher S1P concentrations (5000 nM) only induced fugetaxis, as cells migrated when S1P was added only to lower chamber and did not migrate when S1P was present in both upper and lower chambers (Fig 5B).

### S1P3 is not involved in migration of T-ALL blasts

**Fig 6:**
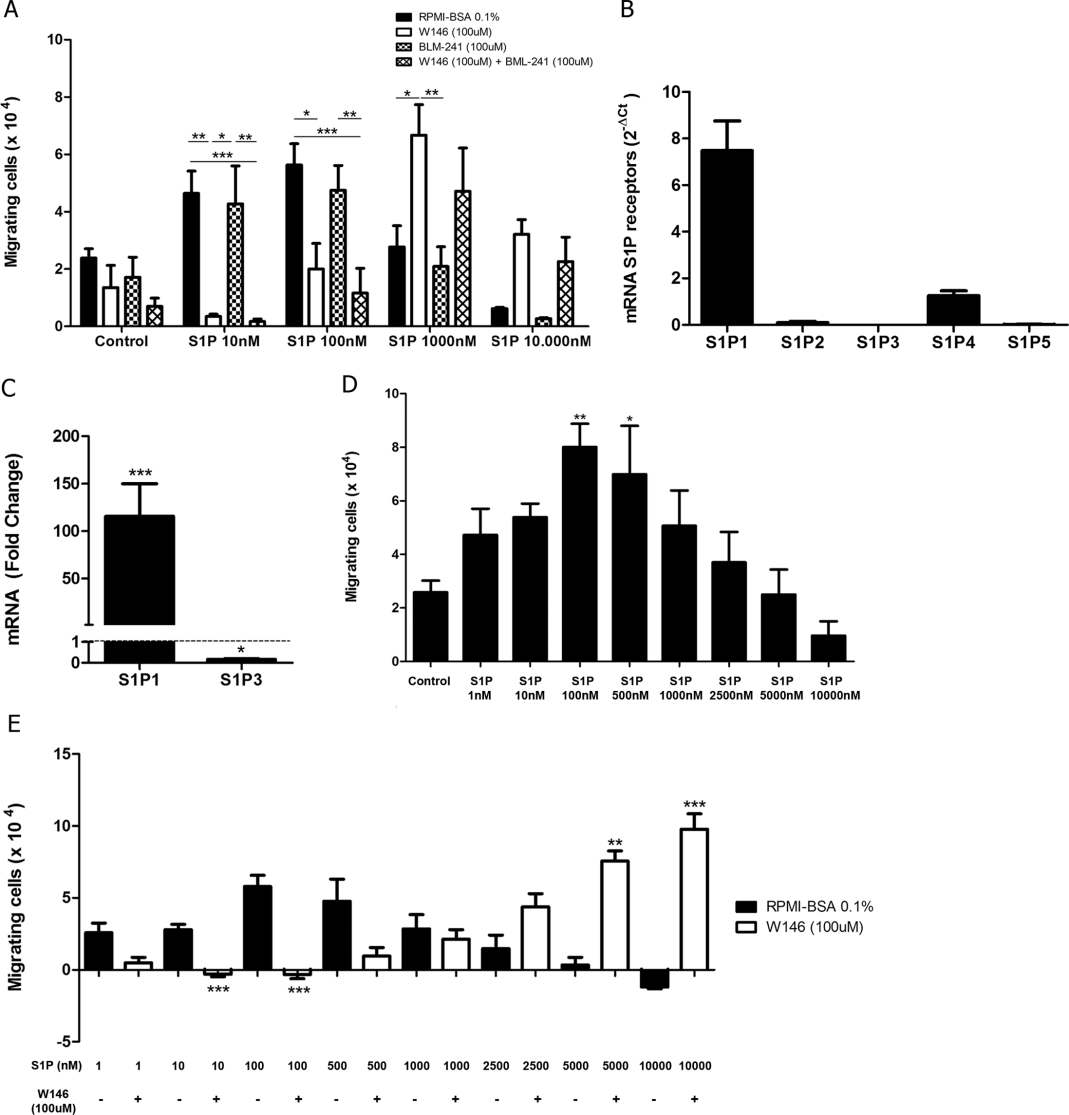
S1P3 is not involved in S1P-driven chemotactic responses of CEM cells. **(A)** CEM cells were serum-starved for 2 h and pre-treated or not with W146 (100 μM) and/or BM-241 (100 μM). Cells were applied in Transwell™ chambers containing different S1P concentrations and incubated for 4 hours. Black bars correspond to pre-treatment with RPMI-BSA 0.1%; white bars correspond to pre-treatment with W146; grid bars correspond to pre-treatment with BML-241; and chess bars correspond to pre-treatment with W146 plus BML-241. Results were analyzed by Two-way ANOVA, followed by Bonferroni post-test (n = 3). **(B)** S1P receptors mRNA expression were analyzed by real time quantitative PCR, compared with the control Abelson (Abl) gene (2^-ΔCt^) in SU-DHL-1 cells (n = 4). **(C)** S1P1 and S1P3 mRNA expression was analyzed by real time quantitative PCR, compared with the control Abelson (Abl). Fold change analysis were done using HPB-ALL as calibrator to normalize the expression of S1P1 and S1P3 on SU-DHL-1 cells. Statistical analysis was made with ΔCt values and significant differences are related to HPB-ALL cells. Results were analyzed by Student’s *t* test (n = 4). **(D)** SU-DHL-1 cells were serum-starved for 2 h, applied to Transwell™ chambers containing different S1P concentrations and incubated for 4 hours. Results were analyzed by One-way ANOVA, followed by Tukey post-test (n = 3). **(E)** SU-DHL-1 cells were serum-starved for 2 h and treated or not with W146 (100 μM). Cells were applied to Transwell™ chambers containing different S1P concentrations and incubated for 4 hours. Values correspond to specific migration after subtracting the numbers of migrating cells in culture medium only. Black bars correspond to pre-treatment with RPMI-BSA 0.1% alone and white bars correspond to pre-treatment with W146. Results were analyzed by unpaired Student’s *t* test (n = 3). Results are expressed as mean ± SEM and differences were considered statistically significant when * p˂0.05, ** p ˂0.01 or *** p ˂0.001.

Migratory responses of CEM cells were completely abrogated following W146 pretreatment in fugetaxis experiments, but not in chemotaxis assays. We therefore hypothesized if other S1P receptor could be activated inducing chemotaxis toward high S1P concentrations following W146 treatment. Besides S1P1, S1P-migrating T-ALL cell lines (MOLT-4, JURKAT and CEM) also expressed S1P3 (Fig 1A–1C), raising the possibility that S1P3 might be involved in cell migratory responses. To evaluate this hypothesis, we performed migration assays with CEM, considering its higher S1P1 expression. We found that pre-treatment of CEM cells with BML-241 (a specific S1P3 blocker) did not inhibit cell migration toward S1P 10 and 100 nM (Fig 6A). In fact, the number of migrating cells in this case was very similar to what was seen when CEM cells were not treated. When CEM cells were pre-treated with W146 and BML-241 together, cell migration was inhibited toward S1P 10 and 100 nM, but did happen when higher S1P concentrations were applied. These data are comparable to those observed when CEM cells were pre-treated only with W146 (Fig 6A), indicating that S1P3 was not involved in cell migration when S1P1 signaling is blocked.

As an additional strategy, we used SU-DHL-1 cells, an anaplasic large cell lymphoma cell line, which expresses S1P1 and S1P4 but not S1P3 (Fig 6B). As compared to HPB-ALL cells, this lymphoma cell line expressed very high levels of S1P1 but even less S1P3 (Fig 6C). As expected, SU-DHL-1 cells were able to migrate toward S1P 100 and 500 nM, but not toward higher S1P concentrations (Fig 6D). Furthermore, when SU-DHL-1 cells were pre-treated with W146, migration toward S1P 10 and 100 nM was inhibited, but was induced toward 5000 and 10000 nM (Fig 6E). Since SU-DHL-1 does not express S1P3 and migration patterns were similar to the ones observed with T-ALL blasts, these results reinforce the notion that S1P3 is not involved in cell migration toward high S1P concentrations under conditions of S1P1 blockage.

### S1P modulates actin polymerization, AKT, ERK and Rac1 activation in T-ALL blasts

**Fig 7:**
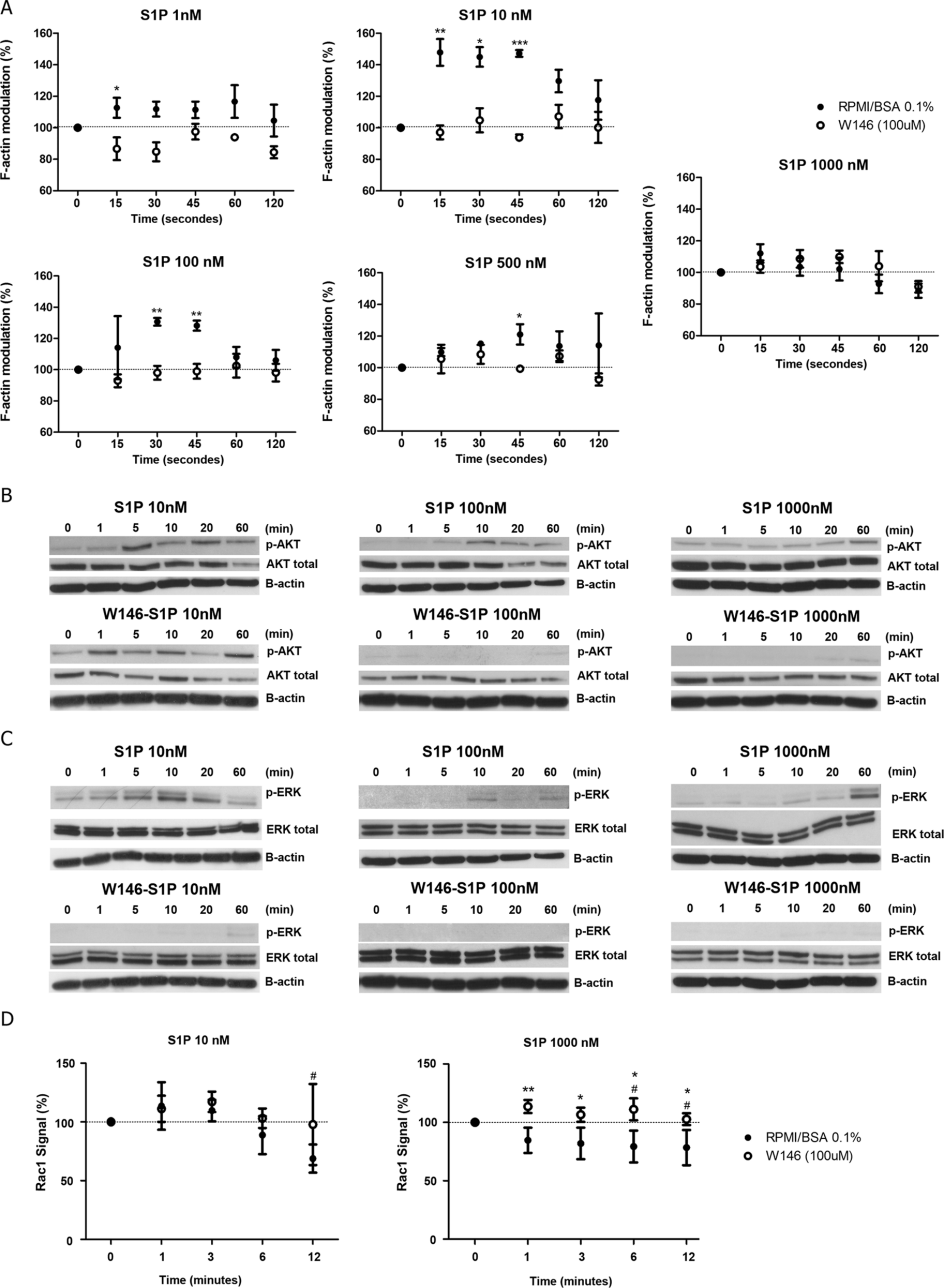
S1P modulates F-actin polymerization, AKT, ERK and Rac1 activity. **(A)** Modulation of the actin cytoskeleton after stimulation by different S1P concentrations in CEM cells pre-treated or not with W146 (100 μM). Results are represented as [(MFI after addition of the ligand) / (MFI before addition of the ligand)] x 100. The MFI values obtained before the addition of S1P were arbitrarily set as 100% and correspond to the time zero. White circles correspond to cells previously treated with W146 (100 μM) and the black circles correspond to cells treated with RPMI-BSA 0.1%. Results are expressed by mean ± SEM and were analyzed by unpaired Student’s *t* test (n = 3). Differences were considered statistically significant when * p˂0.05, ** p ˂0.01 or *** p ˂0.001 **(B)** AKT and **(C)** ERK1/2 activation after stimulation of CEM cells, with different S1P concentrations, being pre-treated or not with W146 (100 μM). Protein extracts were analyzed by Western-blot with AKT, phosphorylated-AKT, ERK1/2 and phosphorylated-ERK-1/2 specific antibodies. Representative western-blots images are shown (n = 3). **(D)** Rac1 activity after stimulation of CEM cells, pre-treated or not with W146 (100 μM), with different S1P concentrations was accessed by G-LISA. Optical density (OD) was detected in 490 nm. Results are represented as [(OD after addition of the ligand) / (OD before addition of the ligand)] x 100. OD values obtained before the addition of S1P were arbitrarily set as 100% and correspond to the time zero. Black circles correspond pre-treatment with RPMI-BSA 0.1% and white circles correspond to pre-treatment with W146 (100 μM). Results and are expressed as mean ± SEM. Differences between distinct time points and control (time zero) were analyzed by One-way ANOVA, followed by Dunnett post-test and were considered statistically significant when # p˂0.05, ## p ˂0.01 or ### p ˂0.001. Differences between pre-treatment with RPMI-BSA 0.1% and pre-treatment with W146 (100 μM) were analyzed by unpaired Student T test (n = 1, with 2 biological replicates in duplicate). Differences were considered statistically significant when * p˂0.05, ** p ˂0.01 or *** p ˂0.001.

Having characterized the S1P modulation of migratory responses of T-ALL blasts, we next analyzed the regulation of actin cytoskeleton, an event necessary for cell migration [33]. As shown in Fig 7A, S1P induced an important actin cytoskeleton polymerization in CEM cells at 10 and 100 nM, the same concentrations that induced chemotactic responses in these cells (Fig 2). Slight polymerization was observed at 1 and 500 nM. Pre-treatment with W146 blocked actin polymerization in all S1P doses applied. Interestingly, the peak of actin polymerization during the kinetics was dose-dependent (Fig 7A).

We then examined AKT, ERK and Rac activation, known to be involved in S1P1 signaling in other cells [34, 35]. After stimulating CEM cells with S1P 10 and 100 nM, which induced cell chemotaxis, we detected a clear AKT phosphorylation at 5 and 10 min, whereas 1000 nM induced a later AKT activation at 60 min (Fig 7B). Cells pre-treated with W146 did not present AKT phosphorylation.

A strong ERK1/2 activation was observed at doses of 10 and 1000 nM (at 10 and 60 min respectively), but only a slight activation at 100 nM. Pre-treatment of CEM cells with W146 also inhibited ERK1/2 activation (Fig 7C).

Despite a slight increase, no significant Rac1 activation was detected when CEM cells were stimulated with S1P 10 nM, whereas a decrease was detected at the end of kinetics (12 minutes post-stimulus) (Fig 7D–left panel). Interestingly, a decrease of Rac1 activity was observed when cells were stimulated with higher S1P concentrations (1000 nM). In contrast, CEM cells pre-treated with W146 presented an increase in Rac1 activation following S1P 1000 nM (Fig 7D–right panel). These data suggest that Rac activation or inhibition can be involved in the particular migratory responses (chemotaxis versus fugetaxis) following different doses of S1P.

## Discussion

The migration of normal and neoplastic T-cell progenitors is guided by a variety of molecules and corresponding receptors. The T-ALL blasts used in this study expressed different amounts of S1P1 mRNA and were able to migrate toward different S1P concentrations. Migration of T-ALL blasts toward S1P 10 nM, a concentration largely used in the literature [18, 19, 36, 37], directly correlated with S1P1 gene expression. This is similar to what we have recently reported for S1P-driven migration of normal human thymocytes [20]. Experimental data in mice also showed that naive CD4^+^ T lymphocytes overexpressing S1P1 migrate more toward S1P 10 nM than wild type CD4^+^ lymphocytes. In addition, the transgenic cells still migrated toward different S1P concentrations when activated with anti-CD3 and anti-CD28, whereas wild type CD4^+^ T cells practically lose their migratory responses, together with a down regulation of S1P1 after activation [36].

In our study, S1P-driven migratory responses of T-ALL cells presented a bell-shaped dose-dependent pattern, with migration increasing from 1 nM to 100 nM and decreasing from 100 to 500 nM. Yet, the curves varied among T-ALL blasts, according to S1P1 gene expression levels. Similar results were described by Iino and colleagues, showing that JURKAT cells migrate toward S1P and the migratory response exhibits a bell-shaped curve [38]. Interestingly, it was reported that CD4 and CD8 L-selectin high single positive thymocytes [19], activated B lymphocytes, transduced with a retrovirus containing a flag-tagged S1P1 insert and a human CD4 reporter, [19, 37] and JURKAT cells [38] lose their migratory response driven by high S1P concentrations (1000 nM).

Our results lead us to investigate whether high doses of S1P1 were inhibiting cell migration (chemotaxis) or were inducing cell repulsion (fugetaxis). We verified that higher concentrations of S1P were actually repulsive to CEM cells and this effect was dependent on S1P1. Interestingly, no CEM chemokinesis was observed at high S1P concentrations, while low S1P concentration induced both chemokinesis and chemoattraction. Dose-dependent chemoattraction versus fugetaxis promoted by the same molecule have been described for chemokines. CXCL12, for example, is attractive to thymocytes when applied at low concentrations and repulsive at high concentrations [39]. IL-8 also induces both migratory processes, being attractive and repulsive to neutrophils [40]. Furthermore, it was described that S1P can be repulsive to osteoclast precursors in high concentrations (10^−6^ M) through S1P2 activation [41], although this effect has never been related to S1P1. The role of S1P2 in migratory responses away from S1P1 was not analyzed in this study since T-ALL blasts expressed very low mRNA levels for this receptor.

In a second vein, it was described, in CCL-23 cells expressing c-myc-tagged S1P1, that W146 treatment resulted in an increase in S1P1 membrane contents probably by inhibiting receptor internalization [42]. In a recent study conducted with B cell lymphoma cells, inhibition of CCR7 endocytosis reverted repulsion to attraction in high CCL19 concentration gradients [43]. The chemoattraction observed in high S1P concentrations after treatment with W146 might correspond to a reversion of migratory orientation.

Different responses of T-ALL blasts toward different S1P concentrations can help to explain previous data described in the literature. Through an experimental model of T-ALL/LBL on zebrafish, Feng and colleagues showed that treatment with W146 inhibited the generation of tumor cell clusters and induced blast dissemination [44]. In addition to the facts discussed above, this may also occur due to a break in S1P gradients, as shown in other studies with different pathologies [45, 46], triggered by an increase in S1P concentrations in the blood stream or in the tumor microenvironment, but also because of S1P-induced chemokinesis. Moreover, upregulation of S1P levels in tumor microenvironment could induce blasts dissemination, as S1P-driven fugetaxis was observed for high S1P concentrations. Therefore, it would be of interest to quantify S1P levels on the bloodstream and tumor of T-ALL and T-LBL in vivo.

SphK1 overexpression it was reported for lymphomas and leukemias, suggesting that these blasts could catalyze S1P production. Tissues from patients with non-Hodgkin’s lymphoma presented higher SphK1 protein and mRNA levels than tissues from patients with reactive lymphoid hyperplasia [47]. Besides that, tumorigenic proerytroblasts presented an upregulation in SphK1 gene expression when compared with nontumorigenic proerytroblasts in a transgenic mouse model of erytroleukemia [48].

Currently, several studies concerning neoplastic cells search to understand cell signaling pathways involved in cell migration processes, since cell motility is directly correlated with invasive and metastatic capacity [49–51]. In addition, specific cell signaling pathways inhibitors have been widely screened for use in the treatment of various malignancies. In this context, we analyzed possible cell signaling pathways activated by S1P/S1P1 interaction in T-ALL blasts. We have previously observed F-actin modulation in normal human T lymphocytes when stimulated with S1P 10 nM; same concentration that induced cell migration upon these cells [20]. As expected, we observed F-actin polymerization in CEM when S1P was used at low concentrations and that modulation was mediated by S1P1. However, we did not observe F-actin modulation when cells were stimulated with high S1P concentrations, suggesting that actin polymerization may occur in a later stage.

S1P/S1P1 interaction can lead to the activation of AKT, ERK, and Rac1 [34, 35]. AKT and ERK activation has been implicated in cell survival and proliferation, respectively. AKT enhances the survival of lymphocytes and others immune cells by inhibiting apoptosis, whereas ERK modulates the proliferation of these cells [34]. In a second vein, it has shown ERK1/2 involvement on S1P-driven migration of DU145 cells, derived from prostate cancer [52]. By contrast, Li and colleagues have reported that ERK was not involved in WiT49 (Wilm’s tumor cell line) cell migration after S1P exposure [27]. Our data shown that low and high S1P concentrations induced AKT and ERK1/2 phosphorylation and S1P1 was involved in the activation of both signaling pathways. CEM cells pre-treated with W146 did not exhibit ERK1/2 phosphorylation when stimulated with S1P, and AKT phosphorylation was prevented when cells were stimulated with high S1P concentrations. Furthermore, when CEM cells were stimulated with high S1P concentrations, AKT and ERK1/2 phosphorylation was seen in a later time point (60 minutes) than in cells stimulated with low S1P concentrations (5–10 minutes). These data are in accordance with our hypothesis that actin polymerization induced by higher S1P concentrations may be occurring in a later stage. It is noteworthy that S1P concentrations used in this work did not alter cell proliferation (until 72 hours—data not shown) and survival (until 48 hours—data not shown and S2 Fig).

The GTPase Rac is required for lymphocyte migration and cell-cell interactions [34, 53, 54]. Modulation of Rac1 activity under high S1P concentrations can be related to the migratory responses observed under the same conditions. CEM cells do not migrate toward high S1P concentrations, when we observed a decrease in Rac1 activity. In contrast, CEM cells pre-treated with W146 expressively migrate toward high S1P concentrations, when Rac1 activity was increased. Previous data indicated a concentration-dependent effect of S1P on Rac and Rho activation. In human pulmonary artery endothelial cells, 500 nM of S1P led to Rac activation, whereas higher S1P concentrations (5 μM) led to Rho activation while occurs a decrease in Rac activation [55]. Induction of distinct signaling pathways was also reported for CXCL12/CXCR4 interactions. Treatment of T-lymphocytes with anti-CXCR4, PTX and Wortmannin (a PI3K inhibitor) prevented chemotaxis and fugetaxis induced by CXCL12, whereas treatment with tyrosine kinase inhibitors (genistein and herbimycin) prevented only the chemotactic migration [56]. Moreover, different calcium and cAMP levels can determine attraction or repulsion in neuron axon guidance [57]. Together these results suggest that chemotaxis and fugetaxis can activate distinct signaling pathways.

As actin polymerization and Rac activation were not observed when CEM cells were stimulated with S1P concentrations related to fugetaxis, another mechanism inducing cell migration, independent of actin cytoskeleton modulation, might be occurring, such as blebs [58]. Indeed, to confirm this hypothesis other signaling pathways involved in the regulation of actin polymerization need to be investigated, including GTPases Cdc42 and Rho [59, 60].

**Fig 8:**
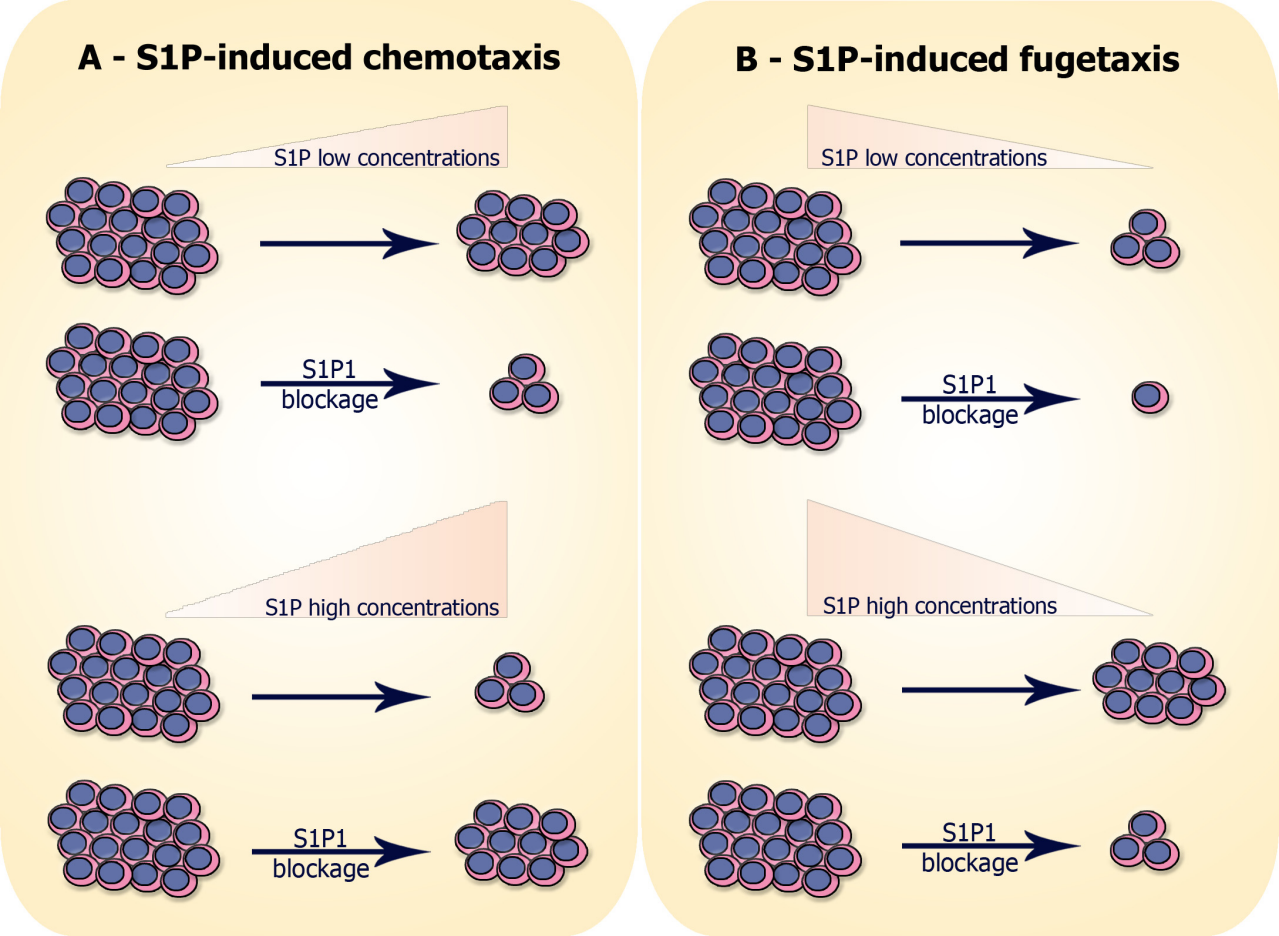
Hypothesis of S1P-induced migration of neoplastic T-cell progenitors: (A) S1P-induced chemotaxis: cells expressing S1P1 migrate toward low S1P concentrations (10–100 nM) and migration is inhibited when S1P1 is blocked with W146. In contrast, cells do not migrate toward high S1P concentrations (500–10000 nM), unless when S1P1 is blocked. (B) S1P-induced fugetaxis: cells expressing S1P1 do not migrate away from low S1P concentrations and the nonspecific migration observed is even smaller when S1P1 is blocked. However, cells migrate away from high S1P concentrations and migration is impaired when S1P1 is blocked.

Signaling pathways involved in cell migration induced by S1P are also involved in other processes important for normal and neoplastic T-cell development, such as cell proliferation and survival [61]. This reinforces the therapeutic potential of targeting S1P-mediated interactions in T-ALL/LBL. For example, the SphK1/SphK2 inhibitor SKi reduces phosphorylated AKT and ERK levels and induces apoptotic death in T-ALL cell lines and primary human patient cells, although some sub-populations are resistant to death. Another SphK2-selective inhibitor (ROMe) do not perturbs these signaling pathways but induces autophagic death of the same cells [62]. In this context, one can imagine that S1P production catalyzed by SphK1/SphK2 could not only promote cell survival through the activation of different signaling pathways but could also induce cell migration, depending on its concentration in the blasts niche. We observed that adding S1P to CEM cell cultures did not change the proliferation or survival rates even in micromolar doses (unpublished data), but S1P induced dose-dependent directed cell migration. We summarize our results on migratory responses of T-ALL blasts resulting in chemotaxis or fugetaxis in Fig 8.

Together, our results shed more light on the dynamics of T-ALL/LBL blasts migration and can benefit the development of therapeutical strategies using S1P and S1P1 blockers. Taking into account the diversity of cellular processes involved in S1P/S1P1 signaling, the strategies may include induction of cell death, diminution of proliferation and prevention of blast dissemination, which can be resultant of chemotaxis or fugetaxis.

## Supporting Information

S1 Fig S1P1 gene expression and S1P-driven migration are directly correlated in T-ALL blasts. **(A)** mRNA expression of S1P1 in T-ALL cell lines. mRNA expression was analyzed by real time quantitative PCR and compared with the control Abelson (Abl) gene (2^-ΔCt^). n = 1–2, with 3–6 biological replicates. **(B)** Cell migration in Transwell™ chambers was analyzed using S1P 10 nM. Values correspond to specific migration after subtracting the numbers of migrating cells obtained for each cell line in wells with culture medium only. Results are expressed as mean ± SEM (n = 3). **(C)** Linear regression of S1P1 mRNA expression and migrating cells.
(TIF)

S2 Fig S1P, W146 and BML-241 are not toxic to T-ALL blasts. After migration assays, **(A)** T-ALL blasts cells **(B)** CEM cells **(C)** SU-DHL-1 cells and again **(D)** CEM cells, but this time blocked with W146 and/or BML-241, that were not able to migrate toward different S1P concentrations, were collected and stained with Anexin-V-APC and propidium iodide; being further analyzed by flow cytometry. Results correspond to relative number (%) of live cells (Anexin-V-APC^-^ PI^-^) and are expressed as mean ± SEM. Black bars correspond to pre-treatment with RPMI-BSA 0.1%; white bars correspond to pre-treatment with W146; grid bars correspond to pre-treatment with BML-241; and chess bars correspond to pre-treatment with W146 plus BML-241. Results are expressed as mean ± SEM and were analyzed by One-way ANOVA, followed by Tukey post-test and by unpaired Student T test (n = 3). Differences were considered statistically significant when * p˂0.05, ** p ˂0.01 or *** p ˂0.001.
(TIF)
