# Supplementary figures and images for: Sphingosine-1-Phosphate Induces Dose-Dependent Chemotaxis or Fugetaxis of T-ALL Blasts through S1P1 Activation
Source: PLoS One. 2016 Jan 29;11(1):e0148137. doi: 10.1371/journal.pone.0148137 (PMC4732661; doi:10.1371/journal.pone.0148137)

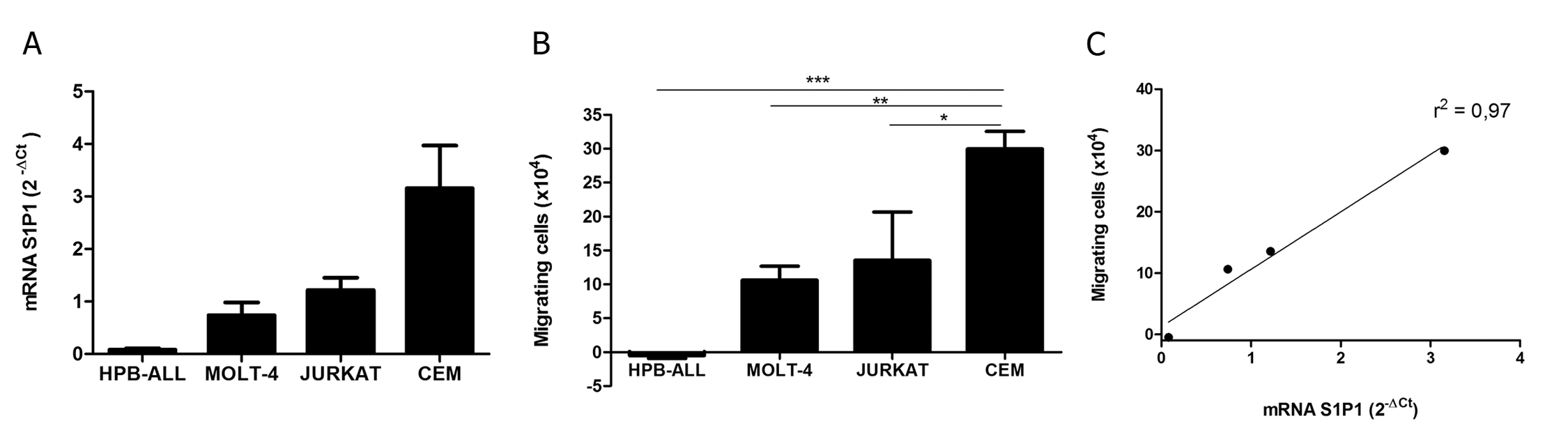

Supplement: S1 Fig — (A) mRNA expression of S1P1 in T-ALL cell lines. mRNA expression was analyzed by real time quantitative PCR and compared with the control Abelson (Abl) gene (2-ΔCt). n = 1–2, with 3–6 biological replicates. (B) Cell migration in Transwell™ chambers was analyzed using S1P 10 nM. Values correspond to specific migration after subtracting the numbers of migrating cells obtained for each cell line in wells with culture medium only. Results are expressed as mean ± SEM (n = 3). (C) Linear regression of S1P1 mRNA expression and migrating cells. (TIF) [file pone.0148137.s001.tif]

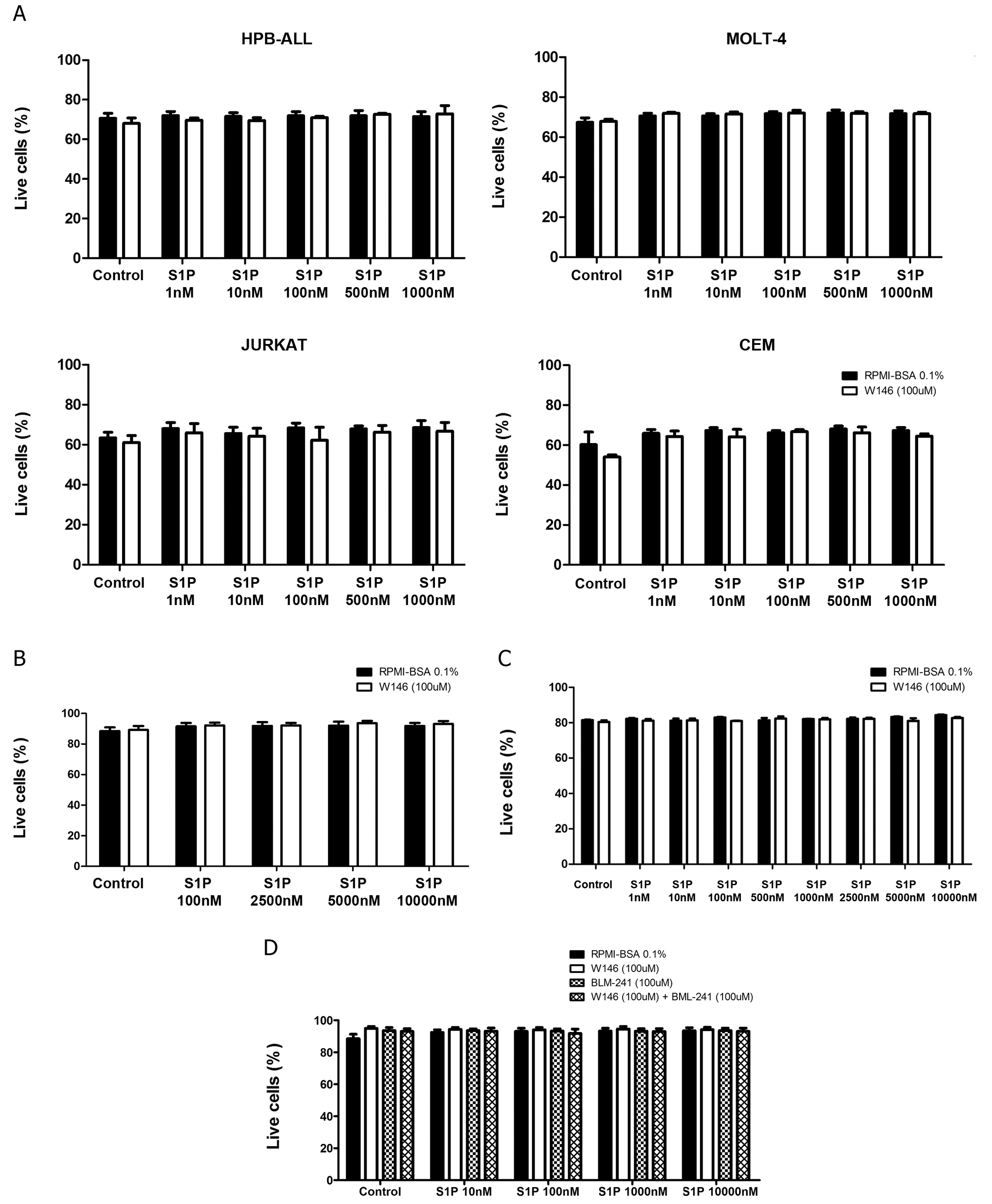

Supplement: S2 Fig — After migration assays, (A) T-ALL blasts cells (B) CEM cells (C) SU-DHL-1 cells and again (D) CEM cells, but this time blocked with W146 and/or BML-241, that were not able to migrate toward different S1P concentrations, were collected and stained with Anexin-V-APC and propidium iodide; being further analyzed by flow cytometry. Results correspond to relative number (%) of live cells (Anexin-V-APC- PI-) and are expressed as mean ± SEM. Black bars correspond to pre-treatment with RPMI-BSA 0.1%; white bars correspond to pre-treatment with W146; grid bars correspond to pre-treatment with BML-241; and chess bars correspond to pre-treatment with W146 plus BML-241. Results are expressed as mean ± SEM and were analyzed by One-way ANOVA, followed by Tukey post-test and by unpaired Student T test (n = 3). Differences were considered statistically significant when * p˂0.05, ** p ˂0.01 or *** p ˂0.001. (TIF) [file pone.0148137.s002.tif]
